# Electroacupuncture ameliorates embryo implantation dysfunction in mice via miR-30c-5p

**DOI:** 10.3389/fendo.2026.1847688

**Published:** 2026-06-02

**Authors:** Jing-Yan Song, Feng-Yi Dong

**Affiliations:** 1 The First Clinical College, Shandong University of Traditional Chinese Medicine, Jinan, China; 2 Reproductive Center of Integrated Medicine, The Affiliated Hospital of Shandong University of Traditional Chinese Medicine, Jinan, China; 3 Department of Obstetrics and Gynaecology, Yong Loo Lin School of Medicine, National University of Singapore, Singapore, Singapore; 4 Child Rehabilitation Center, Jinan Maternity and Child Care Hospital Affiliated to Shandong First Medical University, Jinan, China

**Keywords:** electroacupuncture, embryo implantation dysfunction, exosomes, FAK/JNK pathway, miR-30c-5p

## Abstract

**Objective:**

To investigate the intrinsic mechanism by which electroacupuncture (EA) treats embryo implantation dysfunction in mice from the perspective of exosomal miRNAs.

**Methods:**

Human trophoblast cells (HTR-8/Svneo) were co-cultured *in vitro* with endometrial cells (Ishikawa and HEC-1-A), and exosomes derived from endometrial cells were isolated to treat HTR-8/Svneo cells. Cell proliferation and invasion were assessed by EdU staining and Transwell assays. miRNA sequencing was performed on exosomes from the two endometrial cell lines to identify differentially expressed miRNAs. A mifepristone-induced mouse model of implantation dysfunction was established and treated with EA. The number of implantation sites was counted, pathological changes in the endometrium were observed by hematoxylin-eosin (HE) staining, and miR-30c-5p expression was detected by qPCR. miR-30c-5p was inhibited in HTR-8/Svneo cells, and changes in cell proliferation, invasion, and phosphorylation of FAK and JNK were evaluated.

**Results:**

Communication existed between HTR-8/Svneo cells and endometrial cells, and co-culture with Ishikawa cells significantly promoted the proliferation and invasion of HTR-8/Svneo cells. Exosomes derived from both Ishikawa and HEC-1-A cells promoted HTR-8/Svneo cell proliferation and invasion. High-throughput sequencing showed that miR-30c-5p was expressed at a low level in Ishikawa cell-derived exosomes but at a high level in HEC-1-A cell-derived exosomes. *In vivo* experiments demonstrated that EA reduced miR-30c-5p expression in the endometrium of mice with implantation dysfunction, showed a trend of increasing implantation sites, and alleviated endometrial injury. Inhibition of miR-30c-5p in HTR-8/Svneo cells promoted cell proliferation and invasion and increased the phosphorylation of FAK and JNK.

**Conclusion:**

EA may ameliorate embryo implantation dysfunction in mice by downregulating miR-30c-5p in the endometrium.

## Introduction

Infertility is a global health problem affecting 15% of couples of reproductive age worldwide. In China, the number of individuals with infertility has exceeded 40 million, accounting for 15.5% of married women, and this proportion continues to rise annually (1–3). *In vitro* fertilization-embryo transfer (IVF-ET) is currently a commonly used assisted reproductive technology (4, 5). However, the clinical pregnancy rate (30%-40%) and embryo implantation rate (25%) of IVF-ET remain relatively low, and 10%-15% of women still fail to achieve pregnancy after multiple IVF-ET cycles (6). It is reported that insufficient endometrial receptivity is the major cause of IVF-ET failure (7). Therefore, improving endometrial receptivity may be the key to promoting embryo implantation and treating infertility.

Infertile patients undergoing IVF-ET often choose additional adjuvant therapies to improve pregnancy and live birth rates after embryo transfer (8). Acupuncture treats disease by stimulating specific acupoints either manually or electrically. Its application in IVF-ET can be traced back several decades (9), and increasing evidence indicates that acupuncture can improve indices associated with endometrial receptivity and thereby enhance the clinical pregnancy rate of IVF-ET (10, 11). Acupuncture has become a preferred adjunctive therapy for patients receiving IVF-ET; in the United States, approximately 30% of infertile patients undergo acupuncture before IVF-ET, and this proportion rises to 47% during IVF-ET treatment (12, 13). Several studies have reported that EA increases the number of high-quality embryos and the embryo implantation rate in infertile patients undergoing IVF-ET (14, 15). Our previous work also demonstrated that EA improved IVF-ET pregnancy outcomes in patients with kidney deficiency-associated infertility (16). Although EA has good efficacy, few side effects, and high patient acceptance, current studies have mainly focused on its therapeutic effects, while the underlying mechanisms remain unclear.

A successful pregnancy depends on embryo implantation, a process involving reciprocal interactions between the endometrium and the embryo. An embryo can only be accepted when the endometrium is in a receptive state, known as the window of implantation, which is regulated by maternal hormones (17). During development, the embryo divides to form the inner cell mass and trophoblast cells. Trophoblast cells then undergo spreading, elongation, positioning, and adhesion before implanting into the endometrium (18). To ensure successful implantation, communication between the endometrium and embryonic trophoblast cells is indispensable. However, studies on this process remain limited. Exosomes are cell-derived extracellular vesicles with diameters ranging from 50 to 150 nm (19), and they are widely distributed in biological fluids such as plasma and serum (20). Exosomes have been shown to mediate intercellular communication and regulate physiological functions (21). Embryo-derived exosomes can be taken up by endometrial cells (22), while exosomes present in the culture medium of endometrial cells can also be internalized by trophoblast cells (23). In addition, endometrium-derived exosomes have been shown to enhance the adhesive capacity of HTR-8 trophoblast cells and promote embryo implantation (24). miRNAs are highly conserved small RNAs of approximately 23 nucleotides that regulate gene expression through mRNA silencing and participate in various physiological processes (25). Therefore, elucidating endometrium-trophoblast communication from the perspective of miRNAs is of considerable significance.

In the present study, human trophoblast HTR-8/Svneo cells were co-cultured with human endometrial Ishikawa cells (receptive) and HEC-1-A cells (non-receptive) to investigate the effects of endometrial cells on trophoblast proliferation and invasion. Exosomes were isolated from the supernatants of these two endometrial cell lines and subjected to miRNA sequencing to identify differentially expressed miRNAs. Furthermore, *in vivo* experiments were conducted to explore the effect of EA on miR-30c-5p expression in the endometrium of mice with embryo implantation dysfunction. miR-30c-5p was then inhibited in HTR-8/Svneo cells to assess alterations in cell proliferation and invasion.

## Materials and methods

### Cell culture and treatment

HTR-8/Svneo human trophoblast cells (iCell-h390, iCell Bioscience Inc., China), Ishikawa human endometrial carcinoma cells (CL-0283, Procell, China), and HEC-1-A human endometrial adenocarcinoma cells (IM-H021, Yimo Biosciences, China) were cultured in RPMI-1640 medium (C11875, Gibco, USA), DMEM medium (10566016, Gibco, USA), and McCoy’s 5A medium (PM150710, Procell, China), respectively. All media were supplemented with 10% fetal bovine serum (10099-141, Gibco, USA) and 1% penicillin-streptomycin (G4003, Servicebio, China), and cells were maintained in a humidified incubator at 37 °C with 5% CO_2_.

Ishikawa and HEC-1-A cells were labeled with 5 μM DiI dye (S25170-10mg, Yuanye Bio-Technology, China). After labeling, the cells were collected and co-cultured with HTR-8/Svneo cells in RPMI-1640 medium. For co-culture, Ishikawa cells or HEC-1-A cells and HTR-8/Svneo cells were seeded at a 1:1 ratio in Transwell chambers, with HTR-8/Svneo cells in the lower chamber and Ishikawa or HEC-1-A cells on the upper insert membrane, ensuring contact with the lower culture medium. After 48 h of co-culture, HTR-8/Svneo cells were collected and photographed using a laser confocal microscope.

Exosomes were isolated from Ishikawa and HEC-1-A cells. HTR-8/Svneo cells were treated with 50 μg/mL or 100 μg/mL Ishikawa- or HEC-1-A-derived exosomes for 48 h, and CCK-8 assays were performed to determine the optimal treatment concentration. Subsequently, 50 μg/mL exosomes from Ishikawa or HEC-1-A cells were used to treat HTR-8/Svneo cells for 48 h, after which cells were collected for subsequent experiments.

A specific inhibitor targeting human hsa-miR-30c-5p was designed based on its mature sequence (5’-UGUAAACAUCCUACACUCUCAGC-3’) obtained from the miRDB database (http://mirdb.org/). The miR-30c-5p inhibitor (5’-GCUGAGAGUGUAGGAUGUUUACA-3’) and the negative control (NC) inhibitor (5’-UCUACUCUUUCUAGGAGGUUGUGA-3’) were synthesized by General Biosystems (Anhui) Co., Ltd. (China). HTR-8/Svneo cells were transfected with the respective inhibitors and harvested 48 h post-transfection for downstream analyses.

### EdU staining

Cell proliferation of HTR-8/Svneo cells was evaluated using an EdU Cell Proliferation Detection Kit (C0078S, Beyotime Biotechnology, China). HTR-8/Svneo cells after co-culture or exosome treatment were collected and incubated with EdU working solution prepared in fresh medium for 2 h. After removal of the supernatant, cells were fixed with 4% paraformaldehyde for 15 min and permeabilized with 0.5% Triton X-100 at room temperature for 10 min. Click reaction solution was then added and incubated for 30 min in the dark. Cell nuclei were counterstained with Hoechst 33342, and images were captured under a fluorescence microscope (CKX53, Olympus, Japan).

### Transwell invasion assay

Ishikawa or HEC-1-A cells were co-cultured with HTR-8/Svneo cells for 48 h, or HTR-8/Svneo cells were treated with Ishikawa- or HEC-1-A-derived exosomes for 48 h. HTR-8/Svneo cells were then collected and seeded into the upper chambers of Matrigel-coated Transwell inserts. After incubation for 24 h at 37 °C, the inserts were removed and the medium was discarded. Cells were stained with 0.1% crystal violet for 1 h. Cells remaining on the upper surface were gently removed using cotton swabs. Invaded cells were observed and photographed under a microscope (BX43, Olympus, Japan). Subsequently, the staining solution was removed, 33% acetic acid was added, and absorbance was measured at 562 nm using a microplate reader (WD-2012B, Liuyi Biotechnology, China).

### Exosome isolation and identification

Cell culture supernatants were sequentially centrifuged at 2,000 ×g for 30 min and 10,000 ×g for 45 min at low temperature to remove impurities and large vesicles. The filtrate (0.45 μm) was then ultracentrifuged at 100,000 ×g for 70 min using a Hitachi CP100MX rotor to collect exosomes. The pellet was resuspended in 10 mL PBS, washed, and ultracentrifuged again. The final pellet was resuspended in 100 μL PBS. Samples were allocated as follows: 30 μL for transmission electron microscopy, 10 μL for particle size analysis, 20 μL for nano-flow cytometry, and the remainder was stored at −80 °C. All centrifugation steps were performed at 4 °C.

### High-throughput sequencing of exosomal miRNAs

RNA was extracted from exosomes isolated from the supernatants of Ishikawa and HEC-1-A cells using the miRNeasy Mini Kit (217004, Qiagen, Germany). Reverse transcription was performed using SuperScript™ II Reverse Transcriptase (18064014, Thermo Fisher Scientific, USA) at 50 °C for 1 h followed by 80 °C for 10 min. During library construction, double-stranded synthesis and indexed PCR amplification were performed using Phusion^®^ High-Fidelity DNA Polymerase (M0530L, New England Biolabs, USA). The thermal cycling conditions were as follows: pre-denaturation at 98 °C for 30 s, followed by 10–16 cycles of 98 °C for 10 s, 60 °C for 30 s, and 72 °C for 15 s, with a final extension at 72 °C for 5 min. PCR products were purified and enriched by PAGE, and paired-end sequencing (PE50 mode) was performed on an NovaSeq™ 2500 platform by LCBio (Illumina, USA).

### CCK-8 assay

After treatment of HTR-8/Svneo cells with different concentrations (50 μg/mL and 100 μg/mL) of Ishikawa- or HEC-1-A-derived exosomes for 48 h, 10 μL of CCK-8 reagent (KGA9305, KeyGEN BioTECH, China) was added. Cells were incubated at 37 °C for 2 h, and absorbance was measured at 450 nm using a microplate reader (SuPerMax3100, Flash Spectrum Biotechnology Co., Ltd., China).

### Animal housing and treatment

Eight-week-old female and male SPF-grade KM mice were purchased from SPF Biotechnology Co., Ltd. (license No. SCXK [Jing] 2024-0001, Beijing, China). Mice were housed under controlled conditions at 20–26 °C, 40%–70% humidity, and a 12 h light/12 h dark cycle, with free access to food and water. After 7 days of acclimatization, female mice were randomly assigned to four groups: Control, Model, Model + Progesterone, and Model + EA, with 8 mice in each group. Mice were caged for mating at a female-to-male ratio of 2:1. Vaginal plugs were checked every morning, and the day on which a vaginal plug was observed was defined as day 1 of pregnancy (Day 1).

In the Model group, mifepristone (532933, MedChemExpress, USA) was administered by gavage on Day 4 at 1 mg/mL and 8 mL/kg to establish an embryo implantation dysfunction model. In the Model + EA group, EA treatment was applied from Day 1 to Day 7 at the “Guanyuan” and “Sanyinjiao” acupoints according to the Atlas of Experimental Animal Acupoints, using an EA apparatus (SDZ-III, Hwato, China). During the EA procedure, mice were anesthetized with isoflurane. Acupuncture needles (0.16×13 mm) were inserted into the “Guanyuan” (CV4) and left “Sanyinjiao” (SP6) acupoints. One pair of electrode leads from a single output channel of the electrical stimulator was used to connect the positive electrode clip (red) to the needle at CV4 and the negative electrode clip (white) to the needle at the left SP6, forming a circuit between these two acupoints (**Supplementary Figure 1**). To ensure comprehensive stimulation, the contralateral (right) Sanyinjiao acupoint was alternately stimulated with CV4 on subsequent treatment days. Stimulation was applied using a continuous wave at 2 Hz for 30 min daily. In the Model + Progesterone group, mice received intramuscular injection of 2 mg progesterone as a positive control treatment from Day 1 to Day 7. On Day 8, all female mice were anesthetized, euthanized by exsanguination, and endometrial tissues were collected. The number of pregnant mice was recorded, and implantation sites were counted. All experimental procedures were approved by the Ethics Committee of Jiangxi Zhonghong Boyuan (approval No.: LL-202409130002).

### HE staining

Paraffin-embedded uterine sections were baked, deparaffinized, and rehydrated, followed by staining with hematoxylin solution (ZLI-9610, ZSGB-BIO, China) for 3 min. After rinsing under running water, sections were differentiated in 1% hydrochloric acid alcohol, blued with bluing solution (G1040, Servicebio, China), and stained with eosin (G1100, Solarbio, China) for 3 min. Sections were then dehydrated, mounted, and observed under a microscope (BX43, Olympus, Japan).

### qPCR analysis

Total RNA was extracted from exosomes derived from Ishikawa and HEC-1-A cells and from endometrial tissues using Trizon Reagent (CW0580S, CWBIO, China), and miRNAs were extracted using a miRNA Ultrapure Extraction Kit (CW0627S, CWBIO, China). cDNA was synthesized using a miRNA reverse transcription kit, and quantitative PCR was performed using a real-time PCR system. The reaction conditions were as follows: pre-denaturation at 95 °C for 30 s; denaturation at 95 °C for 10 s; annealing at 58 °C for 30 s; extension at 72 °C for 30 s; for 40 cycles. U6 was used as the internal control, and relative gene expression was calculated using the 2^-ΔΔCt^ method. Primer sequences are listed in **Table 1**.

**Table 1:** Sequences of primers applied in RT-PCR assay.

Gene	Sequences of primers (5’-3’)
U6 F	GCTTCGGCAGCACATATACTAAAAT
U6 R	CGCTTCACGAATTTGCGTGTCAT
miR-30c-5p F	GCGCGTGTAAACATCCTACACT
miR-30c-5p R	AGTGCAGGGTCCGAGGTATT
miR-30c-5p RT	GTCGTATCCAGTGCAGGGTCCGAGGTATTCGCACTGGATACGACGCTGAG
miR-200a-3p F	GCGCGTAACACTGTCTGGTAA
miR-200a-3p R	AGTGCAGGGTCCGAGGTATT
miR-200a-3p RT	GTCGTATCCAGTGCAGGGTCCGAGGTATTCGCACTGGATACGACACATCG
miR-339-5p_R-3 F	CGCGTCCCTGTCCTCCAG
miR-339-5p_R-3 R	AGTGCAGGGTCCGAGGTATT
miR-339-5p_R-3 RT	GTCGTATCCAGTGCAGGGTCCGAGGTATTCGCACTGGATACGACGAGCTC

### Western blotting

After removing the supernatant, cells were lysed with RIPA lysis buffer (C1053, Applygen Technologies Inc., China), centrifuged at 12,000 rpm for 10 min at 4 °C, and the supernatant was collected. Total protein concentration was determined using a BCA Protein Assay Kit (E-BC-K318-M, Elabscience, China). After denaturation, protein samples were separated by SDS-PAGE for 1.5 h and transferred onto PVDF membranes (IPVH00010, Millipore, USA). Membranes were blocked with 5% non-fat milk at room temperature for 1 h and incubated overnight at 4 °C with the following primary antibodies: Mouse anti-β-Actin (HC201, TransGen Biotech, China; 1:2000), p-FAK (3283, Cell Signaling Technology, USA; 1:1000), FAK (AB40794, Abcam, UK; 1:1000), p-JNK (GB12018, Servicebio, China; 1:1000), and JNK (GB114321, Servicebio, China; 1:1000). The next day, membranes were incubated at room temperature for 1 h with HRP-conjugated Goat Anti-Mouse IgG (H+L) (GB23301, Servicebio, China; 1:2000) or HRP-conjugated Goat Anti-Rabbit IgG (H+L) (GB23303, Servicebio, China; 1:2000). Membranes were then incubated with ECL reagent (RJ239676, Thermo Fisher Scientific, USA), and signals were visualized using a chemiluminescence imaging system (Tanon-5200, Tanon Science & Technology Co., Ltd., China).

### Statistical analysis

Statistical analyses and graph generation were performed using GraphPad Prism 8.0 software. Quantitative data are presented as mean ± standard deviation (SD). Differences between two groups were analyzed using the independent-samples t test. Comparisons among multiple groups were performed using one-way analysis of variance (ANOVA), followed by Tukey’s *post hoc* multiple-comparison test. A P value < 0.05 was considered statistically significant.

## Results

### Communication exists between Ishikawa cells and HTR-8/Svneo cells, and Ishikawa cells promote the proliferation and invasion of HTR-8/Svneo cells

**Figure 1:**
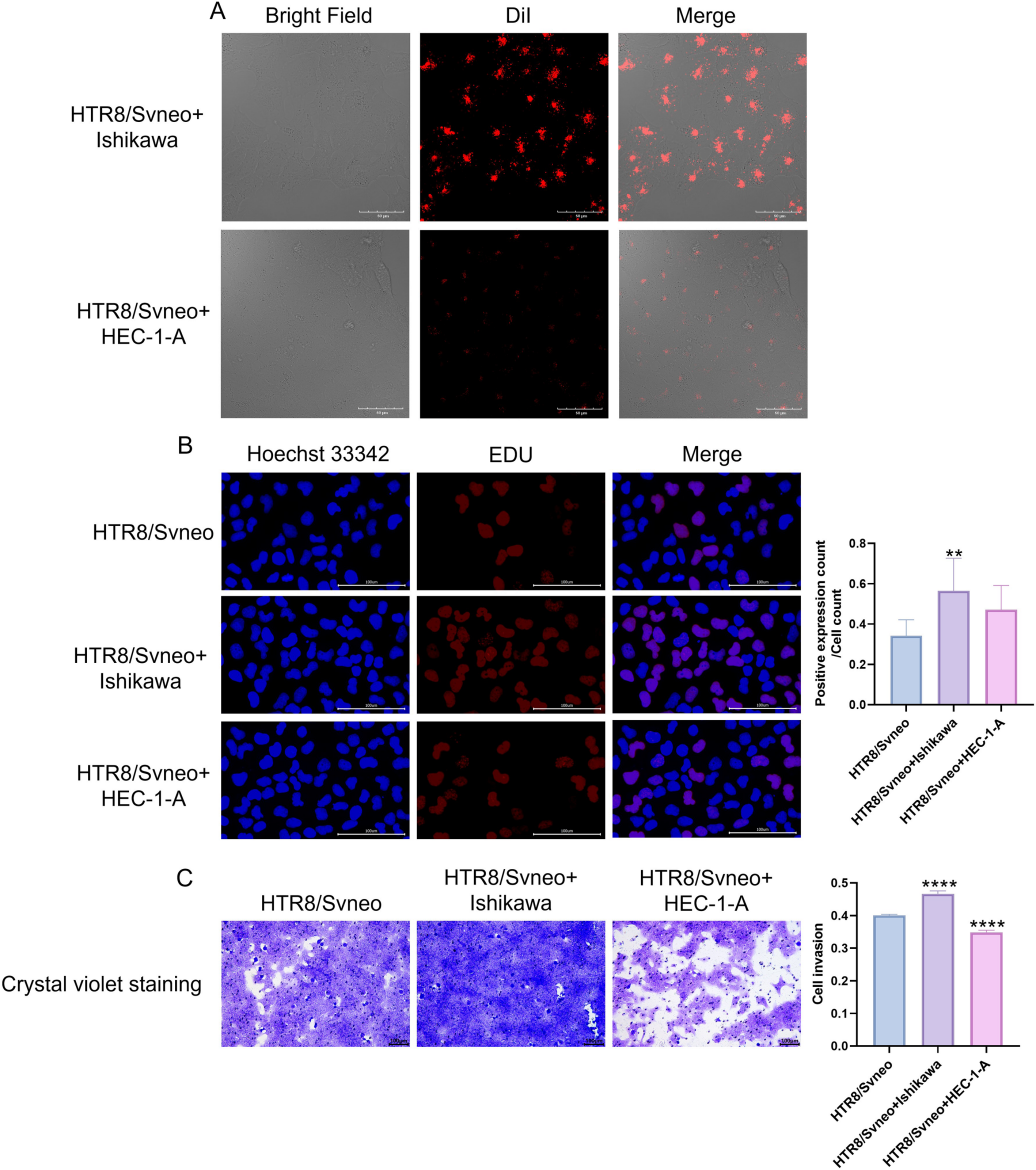
Effects of Ishikawa and HEC-1-A cells on the proliferation and invasion of HTR-8/Svneo cells. **(A)** DiI staining in HTR-8/Svneo cells observed by laser confocal microscopy (scale bar = 50 μm). **(B)** EdU staining for detection of HTR-8/Svneo cell proliferation (scale bar = 100 μm; n = 3). **(C)** Transwell assay for detection of HTR-8/Svneo cell invasion (scale bar = 100 μm; n = 3). **P < 0.01, ****P < 0.0001 vs. HTR-8/Svneo.

HTR-8/Svneo cells were co-cultured with DiI-labeled Ishikawa or HEC-1-A cells, and fluorescence in HTR-8/Svneo cells was observed by laser confocal microscopy. As shown in **Figure 1A**, obvious red fluorescence was detected in HTR-8/Svneo cells after co-culture with Ishikawa cells, whereas only weak red fluorescence was observed after co-culture with HEC-1-A cells, indicating that communication existed between both endometrial cell lines and HTR-8/Svneo cells, with stronger communication between Ishikawa and HTR-8/Svneo cells. EdU staining was performed to assess the effects of Ishikawa and HEC-1-A cells on HTR-8/Svneo cell proliferation. As shown in **Figure 1B**, compared with HTR-8/Svneo cells cultured alone, the number of EdU-positive cells was significantly increased in the Ishikawa + HTR-8/Svneo co-culture group, whereas the HEC-1-A + HTR-8/Svneo co-culture group showed an increasing trend without statistical significance. These results indicate that Ishikawa cells promote HTR-8/Svneo cell proliferation. Transwell assays were conducted to evaluate the effects of Ishikawa and HEC-1-A cells on HTR-8/Svneo cell invasion. As shown in **Figure 1C**, compared with HTR-8/Svneo cells cultured alone, the invasive ability of HTR-8/Svneo cells was significantly increased in the Ishikawa + HTR-8/Svneo co-culture group but significantly decreased in the HEC-1-A + HTR-8/Svneo co-culture group, indicating that Ishikawa cells promote the invasion of HTR-8/Svneo cells.

### Exosomes derived from Ishikawa and HEC-1-A cells promote the proliferation and invasion of HTR-8/Svneo cells

**Figure 2:**
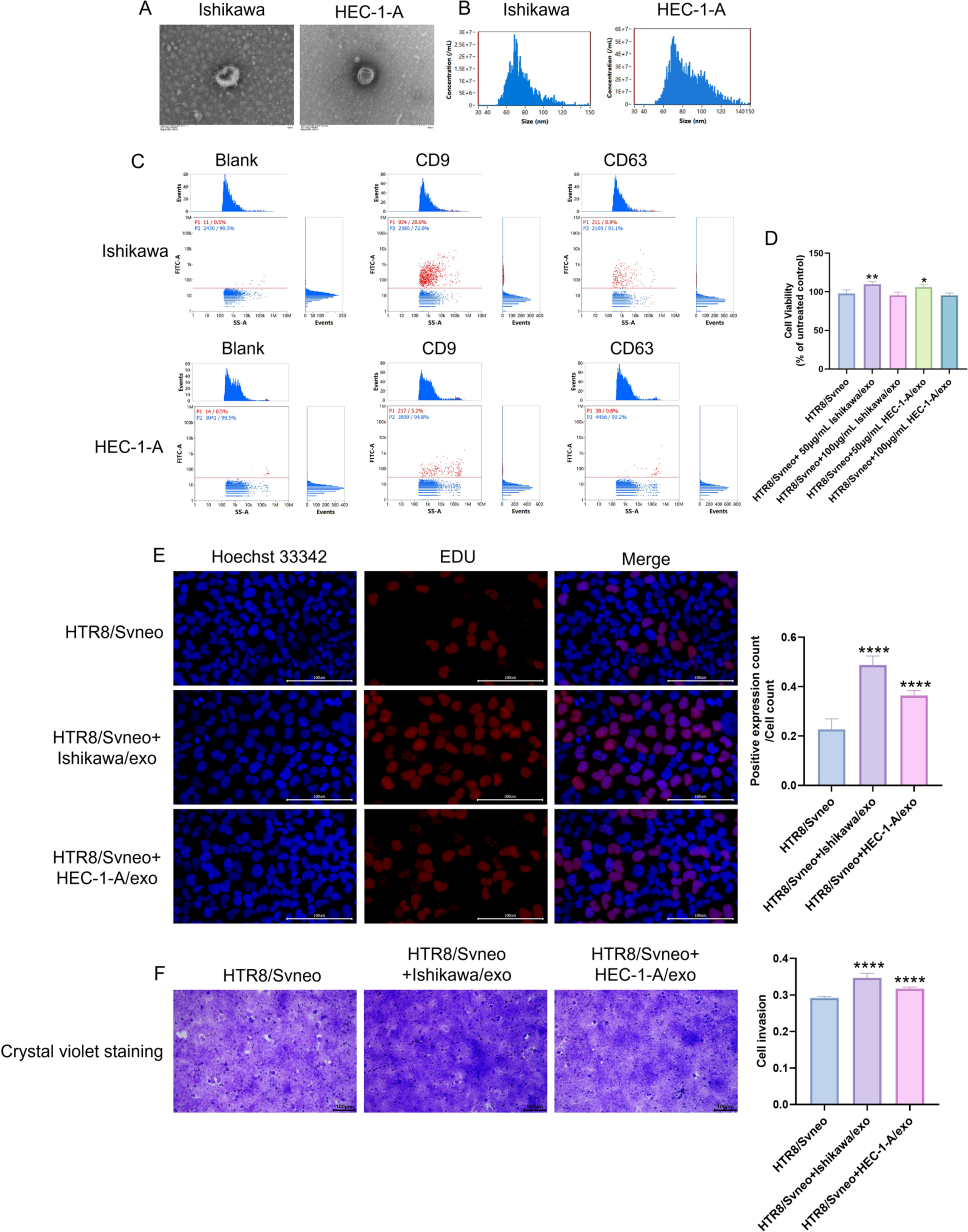
Effects of Ishikawa- and HEC-1-A-derived exosomes on the proliferation and invasion of HTR-8/Svneo cells. **(A)** Exosome morphology observed by transmission electron microscopy (scale bar = 100 nm). **(B)** Nano-flow cytometric analysis of exosome size and concentration. **(C)** Nano-flow cytometric detection of exosomal marker proteins CD9 and CD63. **(D)** CCK-8 assay of HTR-8/Svneo cell viability (n = 3). **(E)** EdU staining for HTR-8/Svneo cell proliferation (scale bar = 100 μm; n = 3). **(F)** Transwell assay for HTR-8/Svneo cell invasion (scale bar = 100 μm; n = 3). *P < 0.05, **P < 0.01, ****P < 0.0001 vs. HTR-8/Svneo.

To determine whether the effects of Ishikawa and HEC-1-A cells on HTR-8/Svneo cell proliferation and invasion were mediated through exosomal communication, supernatants from Ishikawa and HEC-1-A cells were collected for exosome isolation. Exosome morphology was observed by transmission electron microscopy, and particle size, concentration, and expression of exosomal markers (CD9 and CD63) were assessed by nano-flow cytometry. As shown in **Figure 2A**, exosomes derived from both cell types had diameters ranging from 30 to 150 nm and exhibited the typical cup-shaped morphology with a bilayer membrane. As shown in **Figure 2B**, the mean particle size and concentration of Ishikawa-derived exosomes were 76.8 nm and 9.91E + 8 particles/mL, respectively, whereas those of HEC-1-A-derived exosomes were 85.6 nm and 2.80E + 9 particles/mL, respectively. As shown in **Figure 2C**, the positive rates of surface CD9 and CD63 expression were 28% and 8.9%, respectively, in Ishikawa-derived exosomes and 5.2% and 0.8%, respectively, in HEC-1-A-derived exosomes. These results confirmed successful exosome isolation. HTR-8/Svneo cells were treated with different concentrations of Ishikawa- and HEC-1-A-derived exosomes, and cell viability was assessed by CCK-8 assay. As shown in **Figure 2D**, compared with the untreated group, treatment with 50 μg/mL exosomes from either Ishikawa or HEC-1-A cells significantly increased HTR-8/Svneo cell viability. Therefore, this concentration was used in subsequent experiments. EdU staining and Transwell assays further showed that, compared with the untreated group, treatment with either Ishikawa- or HEC-1-A-derived exosomes significantly increased HTR-8/Svneo cell proliferation and invasion, with Ishikawa-derived exosomes exerting a more pronounced effect (**Figures 2E, F**).

### High-throughput sequencing identified differentially expressed miRNAs in exosomes derived from Ishikawa and HEC-1-A cells

**Figure 3:**
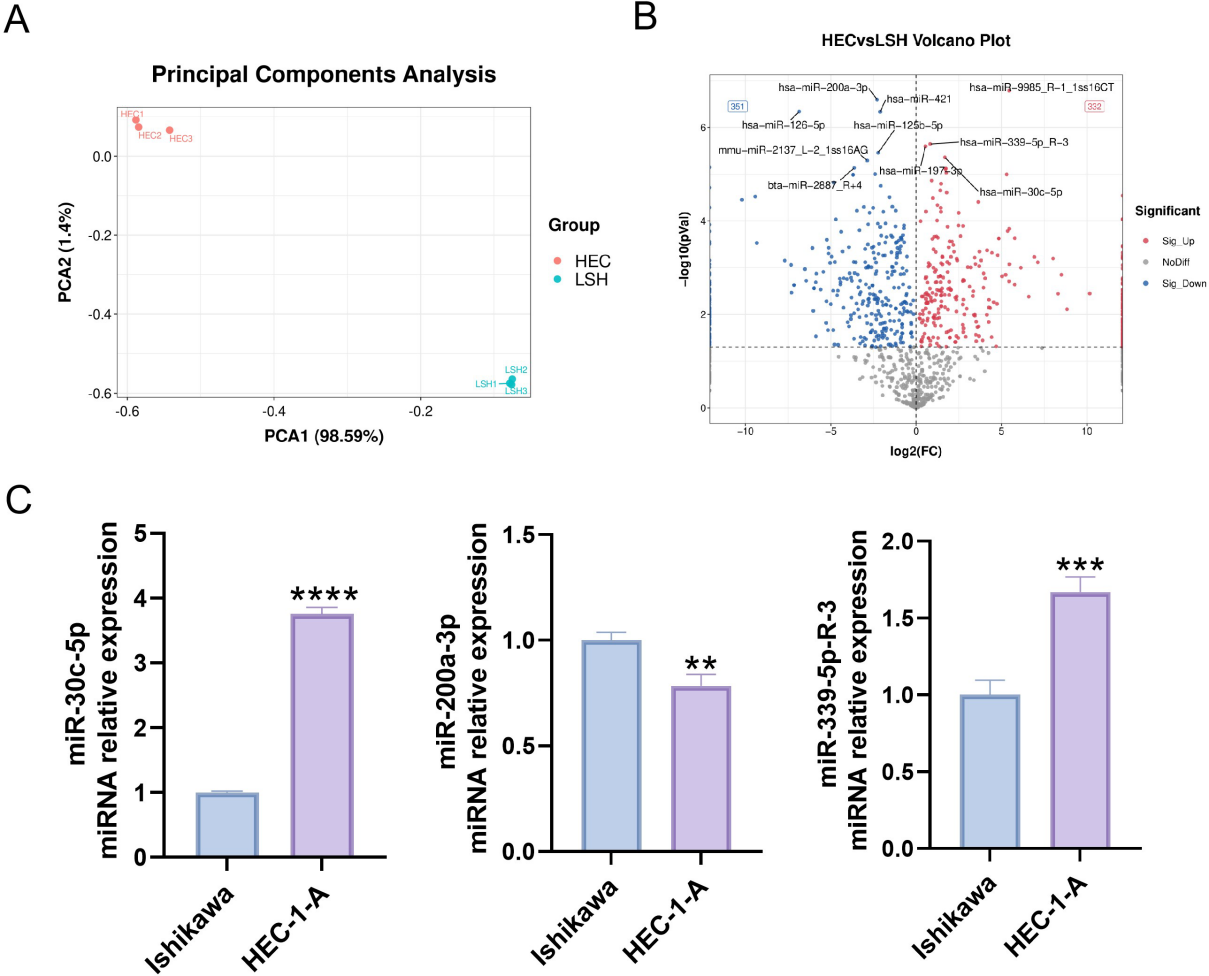
Differential miRNA expression in exosomes derived from Ishikawa and HEC-1-A cells. **(A)** Principal component analysis. **(B)** Volcano plot of differentially expressed miRNAs. **(C)** qPCR analysis of miR-30c-5p, miR-200a-3p, and miR-339-5P-R-3 levels in exosomes derived from Ishikawa and HEC-1-A cells (n = 3). **P < 0.01, ***P < 0.001, ****P < 0.0001 vs. Ishikawa.

The above findings demonstrated that Ishikawa-derived exosomes exerted a stronger promotive effect on HTR-8/Svneo cell proliferation and invasion. To further investigate the underlying mechanism, high-throughput sequencing was performed to identify differentially expressed miRNAs between Ishikawa- and HEC-1-A-derived exosomes. Principal component analysis (PCA) showed that biological replicate samples clustered closely, indicating good reproducibility and consistency (**Figure 3A**). The volcano plot of differentially expressed miRNAs showed that, compared with Ishikawa-derived exosomes, 332 miRNAs were upregulated and 351 miRNAs were downregulated in HEC-1-A-derived exosomes (**Figure 3B**). Three miRNAs among the 10 most significantly altered candidates were selected for qPCR validation. As shown in **Figure 3C**, compared with Ishikawa-derived exosomes, the levels of miR-30c-5p and miR-339-5P-R-3 were significantly increased, whereas the level of miR-200a-3p was significantly decreased in HEC-1-A-derived exosomes. Among these three miRNAs, miR-30c-5p showed the most prominent change and was therefore selected for subsequent experiments.

### Electroacupuncture decreases miR-30c-5p expression in the endometrium and promotes embryo implantation in mice with implantation dysfunction

**Figure 4:**
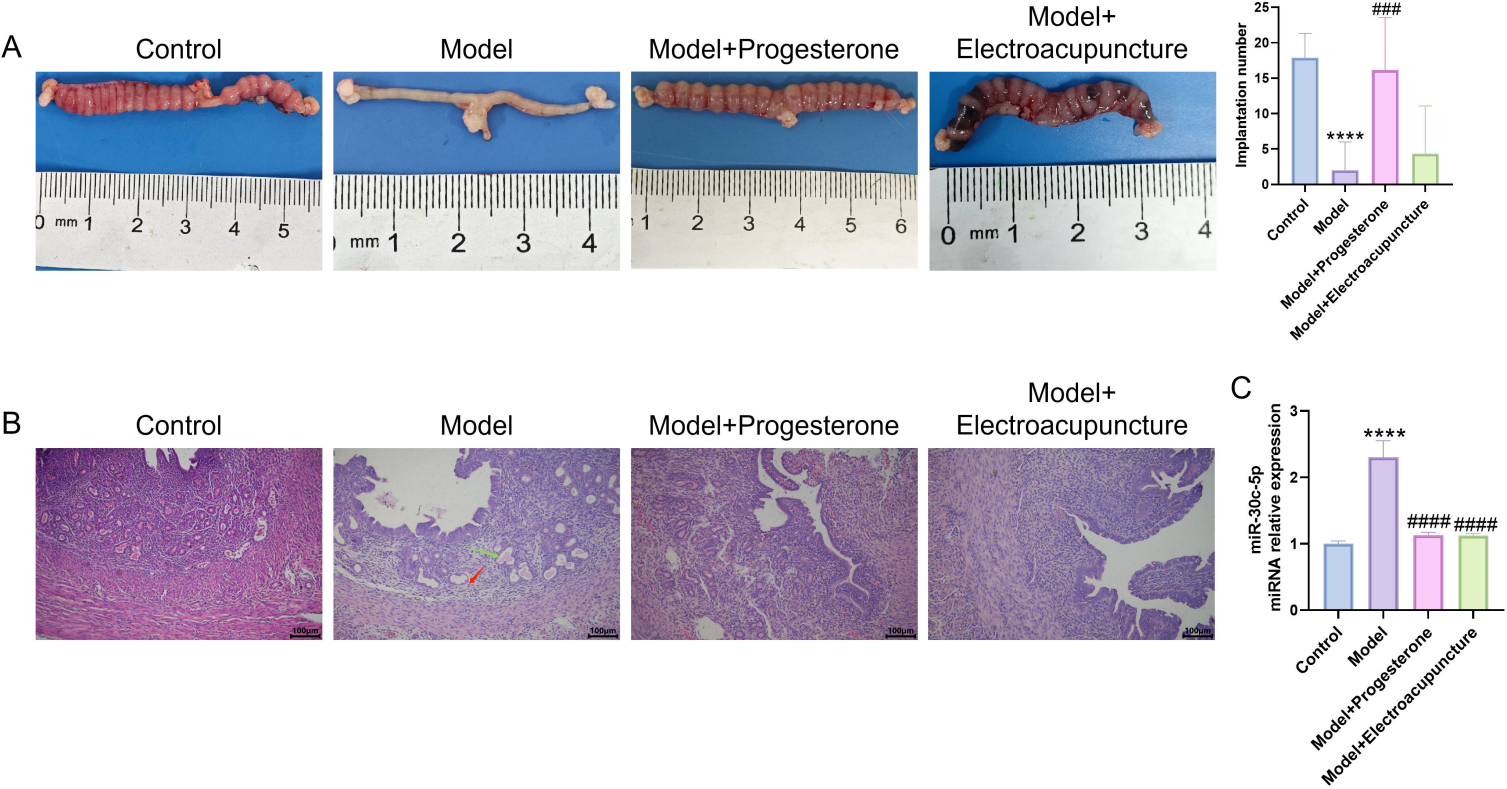
Effects of electroacupuncture on embryo implantation, uterine pathological injury, and miR-30c-5p expression in mice with implantation dysfunction. **(A)** Number of implantation sites in each group (n = 8). **(B)** HE staining showing pathological changes in uterine tissues from each group (scale bar = 100 μm; n = 3). **(C)** qPCR analysis of miR-30c-5p expression in the endometrium of each group (n = 3). ****P < 0.0001 vs. Control; ###P < 0.001, ####P < 0.0001 vs. Model.

A mouse model of implantation dysfunction was established by intragastric administration of mifepristone and treated with EA or progesterone (positive control). As shown in **Figure 4A**, compared with the Control group, the number of implantation sites was significantly reduced in the Model group. Compared with the Model group, the number of implantation sites was significantly increased in the Model + Progesterone group, whereas an increasing trend was observed in the Model + EA group without statistical significance. HE staining was used to evaluate pathological injury in the endometrium of mice from each group. As shown in **Figure 4B**, the uterine cavity in the Control group was clearly dilated, with thickened endometrium, an increased number of glands in the lamina propria, secretions in glandular lumina, densely arranged stromal cells, and proliferated and dilated blood vessels. Compared with the Control group, the Model group exhibited fewer uterine glands with irregular glandular morphology (green arrows) and loosely arranged stromal cells (red arrows). Compared with the Model group, the Model + Progesterone group showed a gradual increase in gland number, marked dilation of glandular lumina, inflammatory cell infiltration, and more densely arranged stromal cells. Compared with the Model group, the Model + EA group showed marked dilation of the uterine cavity, more densely arranged stromal cells, and an increased number of glands. qPCR analysis of miR-30c-5p expression in the endometrium showed that, compared with the Control group, miR-30c-5p expression was significantly increased in the Model group. Compared with the Model group, miR-30c-5p expression was significantly decreased in both the Model + Progesterone and Model + EA groups (**Figure 4C**), suggesting that the therapeutic effect of EA may be associated with downregulation of miR-30c-5p.

### Inhibition of miR-30c-5p promotes the proliferation and invasion of HTR-8/Svneo cells and activates JNK and FAK phosphorylation

**Figure 5:**
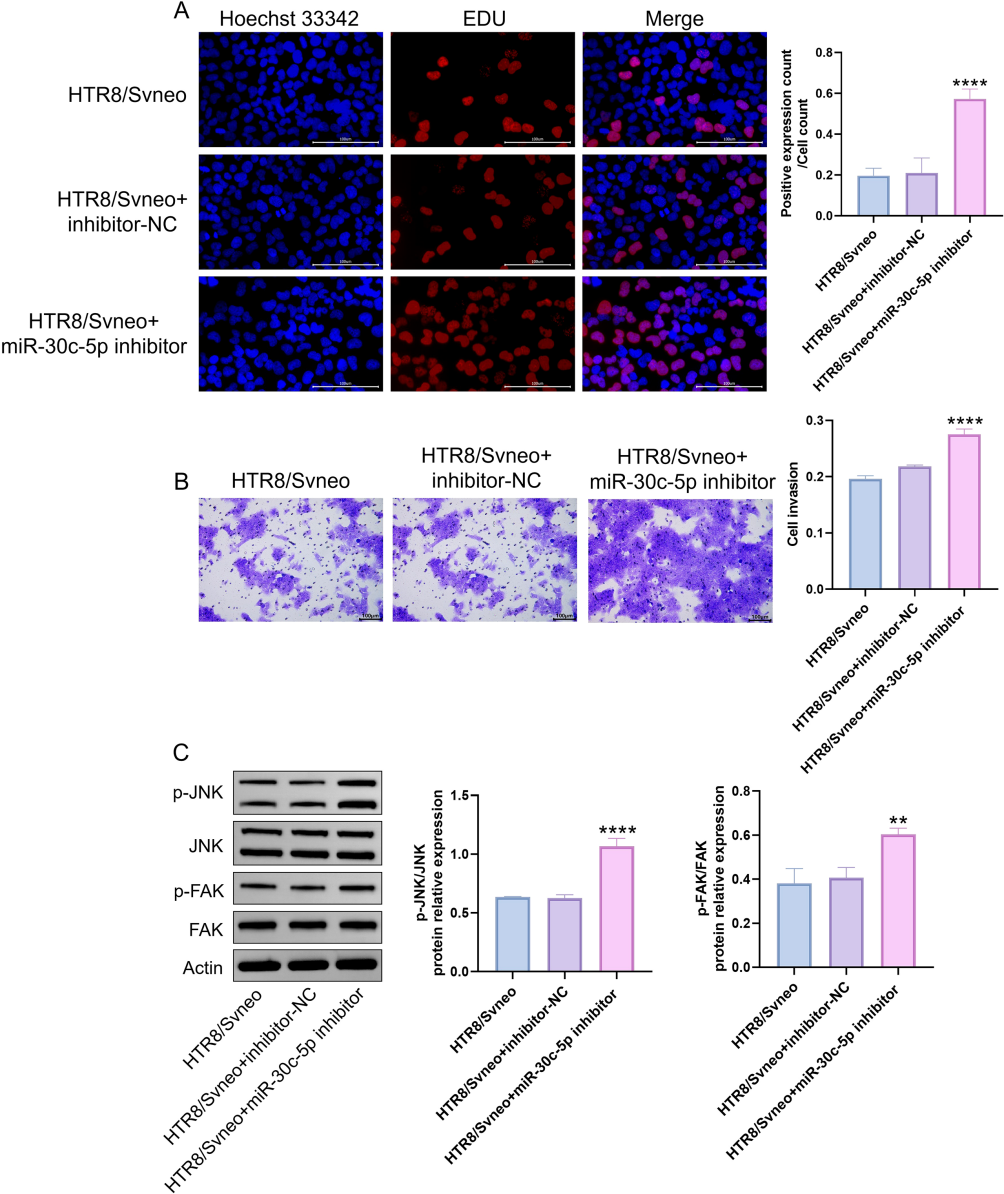
Effects of miR-30c-5p inhibition on the proliferation, invasion, and JNK/FAK phosphorylation of HTR-8/Svneo cells. **(A)** EdU staining for HTR-8/Svneo cell proliferation (scale bar = 100 μm; n = 3). **(B)** Transwell assay for HTR-8/Svneo cell invasion (scale bar = 100 μm; n = 3). **(C)** Western blot analysis of JNK and FAK phosphorylation in HTR-8/Svneo cells (n = 3). **P < 0.01, ****P < 0.0001 vs. HTR-8/Svneo + inhibitor-NC.

To investigate the effect of miR-30c-5p on HTR-8/Svneo cells, HTR-8/Svneo cells were transfected with a miR-30c-5p inhibitor. EdU staining showed that, compared with the HTR-8/Svneo + inhibitor-NC group, the number of EdU-positive cells was significantly increased in the HTR-8/Svneo + miR-30c-5p inhibitor group (**Figure 5A**), indicating that inhibition of miR-30c-5p promoted HTR-8/Svneo cell proliferation. Transwell assays showed that, compared with the HTR-8/Svneo + inhibitor-NC group, the invasive ability of HTR-8/Svneo cells was significantly increased in the HTR-8/Svneo + miR-30c-5p inhibitor group (**Figure 5B**), indicating that inhibition of miR-30c-5p promoted HTR-8/Svneo cell invasion. Western blot analysis further showed that, compared with the HTR-8/Svneo + inhibitor-NC group, the p-JNK/JNK and p-FAK/FAK ratios were significantly increased in the HTR-8/Svneo + miR-30c-5p inhibitor group (**Figure 5C**), indicating that inhibition of miR-30c-5p activated JNK and FAK phosphorylation.

## Discussion

Infertility is a worldwide reproductive health problem, and mature assisted reproductive technologies such as IVF-ET are currently available for treatment. Nevertheless, the embryo implantation rate and clinical pregnancy rate after IVF-ET remain suboptimal. Successful embryo implantation requires bidirectional communication between the endometrium and the embryo. Exosomes are extracellular vesicles with intercellular communication functions and contain a variety of miRNAs, which regulate gene expression by silencing mRNAs. Previous study has shown that the endometrium and embryo can influence each other’s gene expression through exosomal mRNAs, thereby ensuring successful embryo implantation (26). EA is a commonly used adjuvant treatment for IVF-ET and has been shown to enhance endometrial receptivity and improve IVF-ET success rates (27). However, whether EA ameliorates embryo implantation dysfunction by altering miRNA expression in the endometrium remains unclear. In the present study, co-culture of HTR-8/Svneo cells with Ishikawa and HEC-1-A cells showed that both endometrial cell lines communicated with HTR-8/Svneo cells, and Ishikawa cells promoted HTR-8/Svneo cell proliferation and invasion. Exosomes isolated from Ishikawa and HEC-1-A cells also promoted HTR-8/Svneo cell proliferation and invasion. High-throughput sequencing of exosomes derived from Ishikawa and HEC-1-A cells revealed low expression of miR-30c-5p in Ishikawa-derived exosomes, while inhibition of miR-30c-5p in HTR-8/Svneo cells promoted their proliferation and invasion. *In vivo* experiments further demonstrated that EA reduced miR-30c-5p expression in the endometrium of mice with implantation dysfunction, showed a trend of increasing implantation sites, and alleviated endometrial injury.

The co-culture of HTR-8/Svneo cells with DiI-labeled Ishikawa and HEC-1-A cells in the present study also confirmed information exchange between these cell types, with more frequent communication observed in the HTR-8/Svneo and Ishikawa co-culture system. During embryo implantation, trophoblast invasion into the endometrium is a critical step. Villous trophoblasts develop from anchoring villi and migrate into the uterine decidua, replacing arterial endothelial cells and establishing blood supply at the implantation site, thereby facilitating maternofetal exchange (28). It has been reported that the endometrium can regulate trophoblast invasion through matrix metalloproteinases (29). Our co-culture results similarly showed that co-culture with Ishikawa cells promoted HTR-8/Svneo cell proliferation and invasion.

Exosomes are lipid bilayer-enclosed extracellular vesicles with diameters of 50–150 nm and express exosomal markers such as CD9 and CD63 on their surface, enabling them to participate in intercellular communication (30). In the present study, exosomes were isolated from the supernatants of Ishikawa and HEC-1-A cells, and the mean particle size of Ishikawa-derived exosomes was 76.8 nm, while that of HEC-1-A-derived exosomes was 85.6 nm. Both exosome populations expressed CD9 and CD63, indicating successful exosome isolation. It is reported shown that endometrium-secreted exosomes are crucial during the early stage of embryo implantation (31). Endometrium-derived exosomes can be internalized by trophoblast cells and enhance their adhesive capacity (32). Another study similarly reported that exosome treatment enhanced trophoblast migration and improved implantation capacity in an *in vitro* embryo transfer model (33). Consistent with these findings, our results showed that treatment of HTR-8/Svneo cells with Ishikawa- or HEC-1-A-derived exosomes significantly enhanced cell proliferation and invasion, with Ishikawa-derived exosomes exerting a more evident effect. Interestingly, we observed a divergent phenomenon where direct co-culture with HEC-1-A cells inhibited HTR-8/Svneo invasion, whereas purified HEC-1-A-derived exosomes promoted it relative to the control. This discrepancy highlights the complexity of the whole-cell microenvironment versus isolated vesicular fractions. In direct co-culture, HTR-8/Svneo cells are exposed to the entire secretome of HEC-1-A cells, which is widely recognized as a non-receptive endometrial lineage (34). The non-receptive secretome often contains dominating soluble inhibitory cytokines or chemokines that suppress trophoblast invasion. In contrast, purified exosomes intrinsically encapsulate basal lipids, metabolic enzymes, and structural proteins that provide generalized metabolic and survival support to recipient cells (35), thereby leading to a baseline promotive effect compared to untreated (serum-free) controls. Notably, the promotive effect of HEC-1-A exosomes was significantly weaker than that of Ishikawa exosomes. This perfectly aligns with our sequencing data showing that HEC-1-A exosomes are highly enriched in miR-30c-5p, which exerts an inhibitory role on trophoblast functions.

Recent study has shown significant differences in miRNAs contained in endometrial exosomes before and during implantation. miR-34c-5p, miR-210, miR-369-5p, miR-100, and miR-582-5p are highly expressed in exosomes derived from receptive endometrial cells (36). Some of these miRNAs have been confirmed to be associated with embryo implantation. For example, miR-100-5p, which is highly expressed in exosomes from receptive endometrial cells, promotes trophoblast migration and invasion and enhances embryo implantation (37). In the present study, exosomes from Ishikawa and HEC-1-A cell supernatants were subjected to high-throughput sequencing, and 683 differentially expressed miRNAs were identified. Among them, miR-30c-5p, miR-200a-3p, and miR-339-5p-R-3 showed significant differential expression. miR-30c-5p has been reported to be associated with blastocyst development, and increased levels of miR-30c-5p have been detected in spent blastocyst culture medium. Moreover, higher miR-30c-5p levels are associated with a lower likelihood of blastocyst development (38). miR-200a-3p has been predicted to regulate endometrial tissue and preimplantation trophoblast cells (39). Our qPCR validation showed that, compared with HEC-1-A-derived exosomes, Ishikawa-derived exosomes exhibited lower levels of miR-30c-5p and miR-339-5p-R-3 but higher levels of miR-200a-3p, with miR-30c-5p showing the most significant alteration.

High expression of miR-30c-5p not only inhibits blastocyst development but is also associated with preeclampsia, intrauterine growth restriction, and miscarriage (40). miR-30c-5p has also been reported to regulate cell invasion in various diseases. In atherosclerosis, upregulation of miR-30c-5p inhibits oxidized low-density lipoprotein-induced proliferation and migration of vascular endothelial cells (41). miR-30c-5p also suppresses the proliferation, migration, and invasion of esophageal squamous cell carcinoma cells (42). Focal adhesion kinase (FAK) and c-Jun N-terminal kinase (JNK) are kinases associated with cell invasion and adhesion, and their phosphorylation enhances invasive capacity. It has been reported that endometrium-derived exosomes activate FAK and JNK in trophoblast cells after uptake, thereby promoting trophoblast proliferation and invasion (37). In the present study, inhibition of miR-30c-5p increased HTR-8/Svneo cell proliferation and invasion and elevated p-JNK/JNK and p-FAK/FAK levels, indicating that miR-30c-5p inhibition promotes trophoblast proliferation and invasion. EA, as a preferred adjunctive therapy for patients undergoing IVF-ET, has been proven to improve embryo implantation and pregnancy rates. In addition, one study demonstrated that EA enhanced endometrial receptivity and promoted embryo implantation by downregulating miR-223-3p and activating the leukemia inhibitory factor (LIF)/signal transducer and activator of transcription 3 (STAT3) signaling pathway (43). In the present study, a mouse model of embryo implantation dysfunction was established and treated with EA. We found that EA showed a trend of increasing implantation sites and significantly alleviated endometrial pathological injury. More importantly, EA reduced miR-30c-5p expression in the endometrium, suggesting that EA may ameliorate embryo implantation dysfunction by downregulating miR-30c-5p. Although EA treatment significantly alleviated endometrial pathological injury and reduced miR-30c-5p expression, macroscopic implantation sites showed only an increasing trend without reaching statistical significance compared to the model group. This outcome, while partly due to limited sample size, individual variability, and the progressive nature of EA neuromodulation versus direct hormonal replacement, also prompts a deeper neuroanatomical examination of our acupoint selection. The efficacy of EA on internal organs critically depends on somato-autonomic reflexes, requiring segmental overlap between stimulated acupoints and the target organ (44–46). The uterus primarily receives sympathetic preganglionic fibers from T11-L2, which also innervate the endometrium. While Guanyuan (CV4) acupoint (T12/T12-L1 innervation) shows reasonable partial overlap with uterine segments, Sanyinjiao (SP6) (L3-S2 innervation) lacks direct segmental overlap with the core T11-L2 sympathetic segments. This “segmental mismatch” at SP6 may have prevented EA afferent signals from effectively activating uterine autonomic pathways, thus attenuating macroscopic effects on uterine blood flow, endometrial receptivity, and embryo implantation, potentially explaining the non-significant macroscopic results. Recent literature supports selecting acupoints with greater T11-L2 segmental overlap for more potent effects. For instance, a review highlighted Jiaji (EX-B2) or Back-Shu acupoints (e.g., Shenshu BL23, Qihaishu BL24) at T11-L2 segments for more effective regulation of endometrial function via somato-autonomic reflexes (47). Future investigations should explore these more segmentally-matched acupoints. Nevertheless, our findings of significant microscopic endometrial injury alleviation and miR-30c-5p downregulation confirm EA’s notable biological impact even with the current acupoint selection strategy.

We acknowledge several limitations in the present study. First, due to the extreme technical challenges of avoiding severe mechanical trauma to the uterine cavity during the highly sensitive “window of implantation”, we did not perform *in vivo* direct intra-uterine injection of miR-30c-5p antagomirs or establish endometrium-specific knockout mouse models. Such procedures could independently cause implantation failure, thereby confounding the causal relationship. Second, the extremely small size of the early-pregnancy mouse uterus limits the yield of *in vivo* tissue-derived exosomes, preventing us from treating *in vitro* human trophoblasts directly with exosomes isolated from EA-treated mice. To navigate these technical barriers, we employed human endometrial cell lines as an *in vitro* proxy to mimic maternal-fetal crosstalk. Future studies utilizing non-invasive targeted delivery systems, such as adeno-associated virus (AAV) vectors, are warranted to directly validate the *in vivo* necessity and sufficiency of the EA/miR-30c-5p axis.

## Conclusion

In conclusion, the present study revealed the effect of miR-30c-5p in endometrial exosomes on trophoblast proliferation and invasion and suggested that EA may improve embryo implantation dysfunction by reducing miR-30c-5p expression. These findings provide a novel perspective for understanding the therapeutic mechanism of EA in infertility treatment.

## Data Availability

The original contributions presented in the study are included in the article/**Supplementary Material**. Further inquiries can be directed to the corresponding author.

## Supplementary material

Supplementary Figure 1 Representative image of the electroacupuncture procedure in mice. The mouse is under isoflurane anesthesia. A red alligator clip connects a needle at the abdominal Guanyuan (CV4) acupoint, and a white alligator clip connects a needle at the left Sanyinjiao (SP6) acupoint, forming a single electrical stimulation circuit.
