# Supplementary figures and images for: Electroacupuncture ameliorates embryo implantation dysfunction in mice via miR-30c-5p
Source: Front Endocrinol (Lausanne). 2026 Jun 2;17:1847688. doi: 10.3389/fendo.2026.1847688 (PMC13268930; doi:10.3389/fendo.2026.1847688)

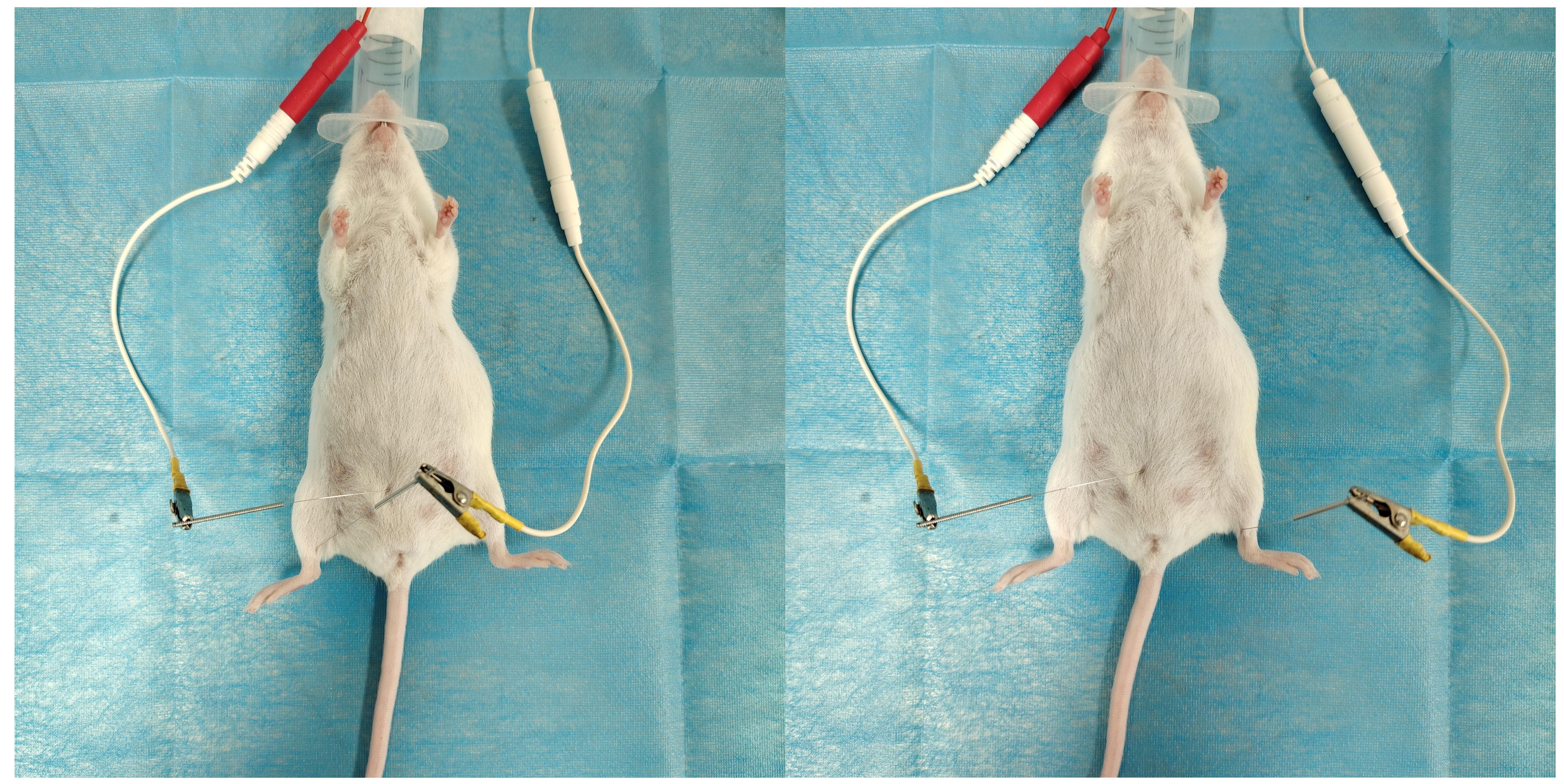

Supplement: Supplementary Figure 1 — Representative image of the electroacupuncture procedure in mice. The mouse is under isoflurane anesthesia. A red alligator clip connects a needle at the abdominal Guanyuan (CV4) acupoint, and a white alligator clip connects a needle at the left Sanyinjiao (SP6) acupoint, forming a single electrical stimulation circuit. [file Image1.jpeg]

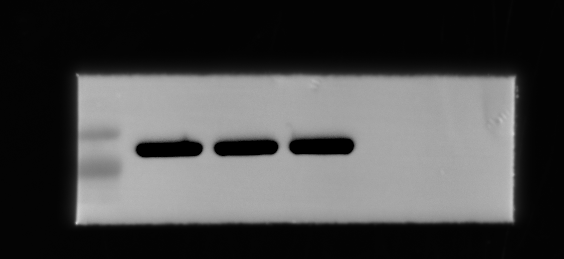

Supplement: Supplementary file 2 [file DataSheet1.zip › ACTIN/ACTIN 1.tiff]

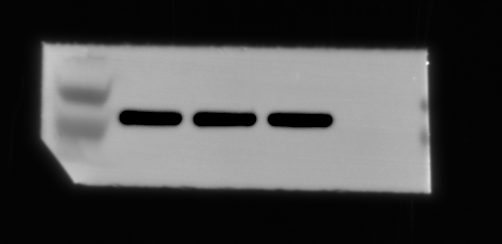

Supplement: Supplementary file 2 [file DataSheet1.zip › ACTIN/ACTIN 2.tiff]

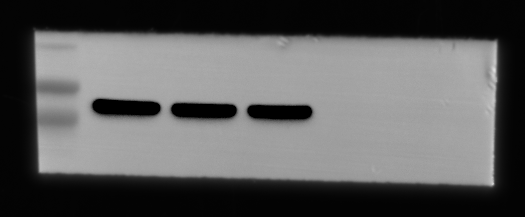

Supplement: Supplementary file 2 [file DataSheet1.zip › ACTIN/ACTIN 3.tiff]

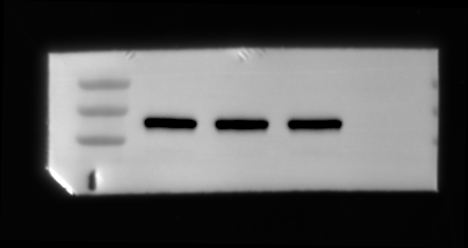

Supplement: Supplementary file 2 [file DataSheet1.zip › FAK/FAK 1.tiff]

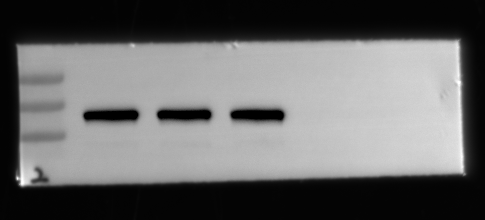

Supplement: Supplementary file 2 [file DataSheet1.zip › FAK/FAK 2.tiff]

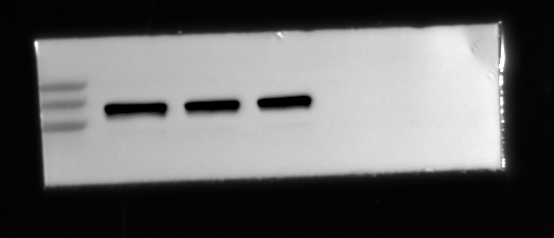

Supplement: Supplementary file 2 [file DataSheet1.zip › FAK/FAK 3.tiff]

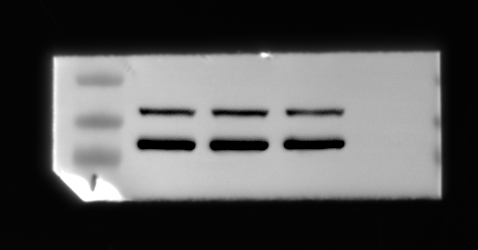

Supplement: Supplementary file 2 [file DataSheet1.zip › JNK/JNK 1.tiff]

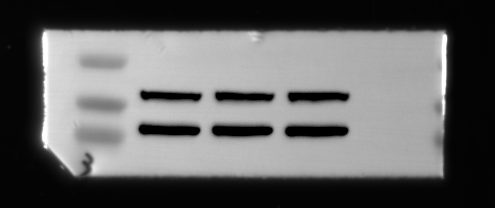

Supplement: Supplementary file 2 [file DataSheet1.zip › JNK/JNK 2.tiff]

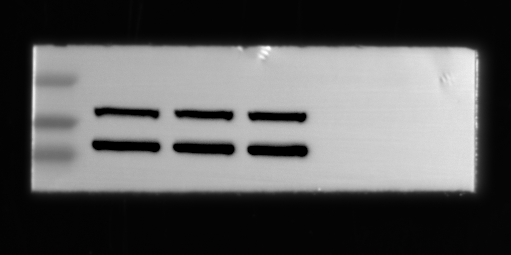

Supplement: Supplementary file 2 [file DataSheet1.zip › JNK/JNK 3.tiff]

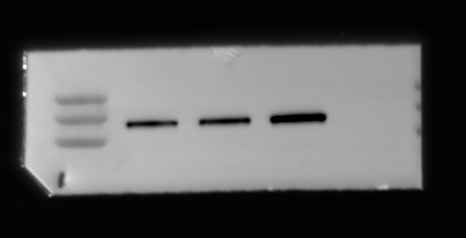

Supplement: Supplementary file 2 [file DataSheet1.zip › P-FAK/P-FAK 1.tiff]

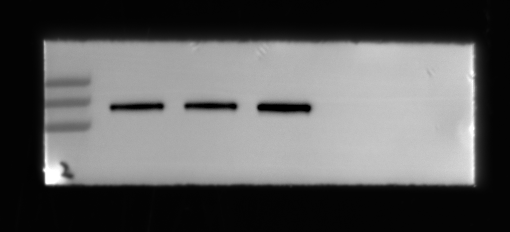

Supplement: Supplementary file 2 [file DataSheet1.zip › P-FAK/P-FAK 2.tiff]

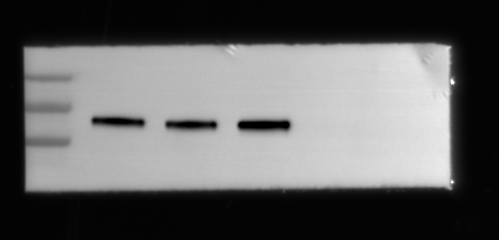

Supplement: Supplementary file 2 [file DataSheet1.zip › P-FAK/P-FAK 3.tiff]

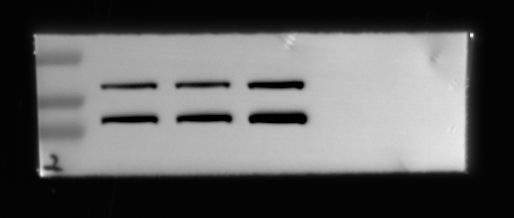

Supplement: Supplementary file 2 [file DataSheet1.zip › P-JNK/P-JNK 1.tiff]

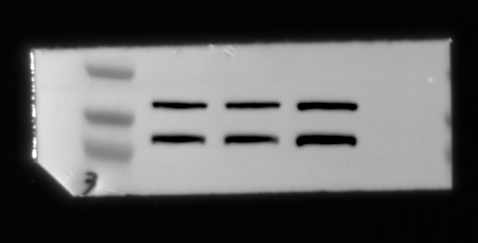

Supplement: Supplementary file 2 [file DataSheet1.zip › P-JNK/P-JNK 2.tiff]

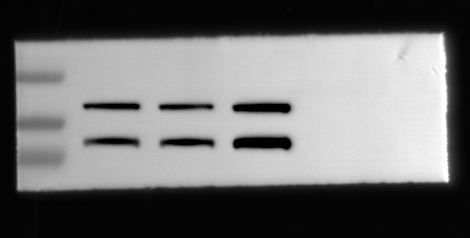

Supplement: Supplementary file 2 [file DataSheet1.zip › P-JNK/P-JNK 3.tiff]
